# Remifentanil ameliorates intestinal ischemia-reperfusion injury

**DOI:** 10.1186/1471-230X-13-69

**Published:** 2013-04-22

**Authors:** Steven SC Cho, Ina Rudloff, Philip J Berger, Michael G Irwin, Marcel F Nold, Wei Cheng, Claudia A Nold-Petry

**Affiliations:** 1 The Ritchie Centre, Monash Institute of Medical Research, Monash University, Melbourne, Australia; 2 Department of Anaesthesiology, University of Hong Kong, Hong Kong, China; 3 Department of Paediatric Surgery, Monash Children’s, Southern Health, Melbourne, Australia; 4 Department of Paediatrics and Department of Surgery, Southern Clinical School, Faculty of Medicine, Nursing and Health Sciences, Monash University, Melbourne, Australia

**Keywords:** Ischemia, Reperfusion, Intestine, Mouse, Opioid, Remifentanil, Preconditioning

## Abstract

**Background:**

Intestinal ischemia-reperfusion injury (IRI) can occur in clinical scenarios such as organ transplantation, trauma and cardio-pulmonary bypass, as well as in neonatal necrotizing enterocolitis or persistent ductus arteriosus. Pharmacological protection by pretreating (“preconditioning”) with opioids attenuates IRI in a number of organs. Remifentanil appears particularly attractive for this purpose because of its ultra-short duration of action and favorable safety profile. To date, little is known about opioid preconditioning of the intestine.

**Methods:**

Young adult C57BL/6J mice were randomly assigned to receive tail vein injections of 1 μg/kg of remifentanil or normal saline and underwent either ischemia-reperfusion of the intestine or a sham laparotomy. Under isoflurane anesthesia, the mice were subjected to intestinal ischemia-reperfusion by occlusion (clamping) of the superior mesenteric artery for 30 min, followed by unclamping and 60 min of reperfusion. After completion of this protocol, tissue injury and lipid peroxidation in jejunum and ileum were analyzed by histology and malondialdehyde (MDA), respectively. Systemic interleukin (IL)-6 was determined in the plasma by ELISA.

**Results:**

Pretreatment with remifentanil markedly reduced intestinal IRI (*P* < 0.001): In the ileum, we observed a more than 8-fold decrease in injured villi (4% vs 34% in saline-pretreated animals). In fact, the mucosa in the remifentanil group was as healthy as that of sham-operated animals. This protective effect was not as pronounced in the jejunum, but the percentage of damaged villi was still reduced considerably (18% vs 42%). There was up to 3-fold more tissue MDA after intestinal ischemia-reperfusion than after sham laparotomy, but this increase in lipid peroxidation was prevented by preconditioning with remifentanil (*P* < 0.05). The systemic inflammatory response triggered by intestinal IRI was significantly attenuated in mice pretreated with remifentanil (159 vs 805 pg/ml of IL-6 after saline pretreatment, with 92 pg/ml in the sham groups). After sham operations, no difference was detected between the saline- and remifentanil-pretreatments in any of the parameters investigated.

**Conclusion:**

Preconditioning with remifentanil attenuates intestinal IRI and the subsequent systemic inflammatory response in mice. We therefore suggest that prophylaxis with this ultra-short-acting opioid may be advantageous in various clinical scenarios of human IRI.

## Background

Interruption of blood supply to an organ rapidly leads to cellular dysfunction and cell death [1]. Although subsequent reperfusion can salvage affected tissues after sustained ischemia, the reperfusion itself induces further injury. Ischemia-reperfusion injury (IRI) can be attributed to many factors such as the release of free oxygen radicals and consecutive lipid peroxidation, cell death by apoptosis or necrosis, inflammatory cytokines, and damage to the microvasculature [1-4].

Preconditioning refers to application of a protective intervention prior to ischemia. This can be mechanical (brief episodes of ischemia and reperfusion) or pharmacological. Ischemic preconditioning was first proposed as a possible therapeutic strategy in 1986, where it was shown to reduce infarct sizes in rat hearts by up to 75% [5]. Since then, ischemic preconditioning has been tested and demonstrated to attenuate IRI in other organs such as the lung [6], kidney [7], liver [8], brain [9], spinal cord [10], and intestine [11].

However, the application of mechanical preconditioning is often difficult or, particularly in the human, impossible. Therefore, interest in pharmacological preconditioning has increased, and opioids were among the first drugs shown to trigger the cascades of preconditioning. In fact, it is interesting to note that the primary use of several of the drugs capable of preconditioning is in perioperative anesthesia (opioids and volatile sedatives [12-16]). Other examples of agents include nitric oxide and glutamine supplementation as well as the use of antioxidants and leptin [1,17].

IRI of the intestine can occur in many medical and surgical situations such as cardiopulmonary bypass, trauma, organ transplantation, as well as in neonatal diseases, including necrotizing enterocolitis and persistent ductus arteriosus [18,19]. Ischemia-reperfusion to the intestine is detrimental not only for nutrient absorption by the mucosa but also compromises barrier integrity, leading to possible bacterial translocation across the gut wall, peritonitis and subsequently systemic sepsis [18].

Remifentanil is an ultra-short acting, synthetic μ-opioid analgesic with an onset of action of only 1 minute. Its half-life is also very short (3 to 5 minutes) as it is rapidly degraded by tissue and blood esterases. These unique pharmacokinetic properties of remifentanil make it an easily manageable preconditioning agent. Studies into the benefits of remifentanil-induced preconditioning have to date been limited to the heart, liver and kidney [20-25]. Here, we set out to investigate the possible protective effects of remifentanil on intestinal IRI. Using an adult mouse model of intestinal IRI, we assessed the protective properties of remifentanil by analyzing the degree of injury and oxidative stress by histology and malondialdehyde (MDA) assays, as well as the systemic effects by measuring interleukin 6 (IL-6).

## Methods

### Animals

Young male adult (8–12 weeks) C57BL/6J mice weighing 24 to 30 g were used for this study. Mice were obtained from the Monash Medical Centre Animal Facility (Monash Medical Centre, Melbourne, Australia) and reared at room temperature on a 12 h/12 h light–dark cycle in specific pathogen-free housing prior to experimentation. They were fed standard rodent diet with free access to water. All animal work conformed with the guidelines established by the National Health and Medical Research Council of Australia and had the approval of the Standing Committee in Ethics in Animal Experimentation of Monash University.

### Ischemia-reperfusion model

Mice were anesthetized with 2% isoflurane (balance O_2_) by inhalation through a gas mask. All animals were secured in a supine position onto a heating pad to maintain normal body temperature during the procedure. Mice were first randomly separated into a sham and an ischemia-reperfusion group. Within each group, the animals were then again randomly allocated into two groups to receive a 300 μl slow (1 min) tail vein injection of either: (1) vehicle control (normal saline) or (2) 1 μg/kg remifentanil (Ultiva; GlaxoSmithKline Limited, Melbourne, Australia). Five minutes after injection, a lower midline laparotomy was performed, the superior mesenteric artery (SMA) identified and clamped with a non-crushing clamp for 30 minutes. Thereafter, the SMA was unclamped, by which a 60 minute period of reperfusion commenced. During the ischemia-reperfusion period, the mice continuously received 1% isoflurane in O_2_. At the end of the reperfusion period, blood was collected via cardiac puncture. All animals were humanely killed via cervical dislocation.

### Histological analysis

**Figure 1:**
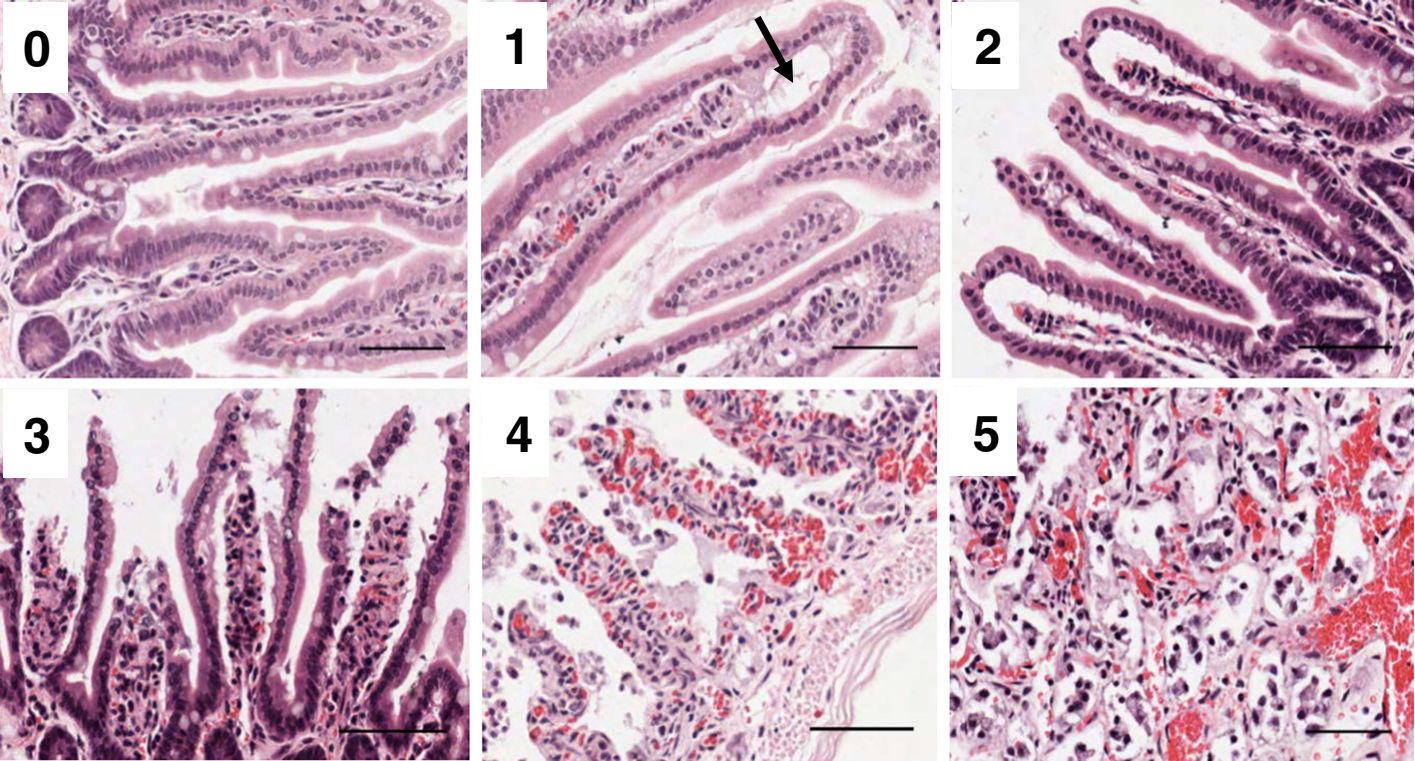
**Blueprint of the histological grading scheme of the increasing severity of ischemia-reperfusion-induced intestinal mucosal lesions.** As described by Chiu [26], we graded the intestinal injury as follows: **0,** normal villi; **1**, development of subepithelial Gruenhagen’s space (black arrow) usually at the apex of the villus, often accompanied by capillary congestion; **2**, extension of the subepithelial space with moderate lifting of epithelial layer from lamina propria; **3**, marked epithelial lifting down the sides of the villi, a few tips may be denuded; **4**, denuded villi with lamina propria and dilated capillaries exposed, increased cellularity of lamina propria may be seen; **5**, digestion and disintegration of the lamina propria, hemorrhage and ulceration. Magnification 200 ×; scale bars indicate 50 μm.

The small intestine was collected and flushed with ice-cold phosphate buffered saline (PBS) to remove intraluminal contents. Tissue was evenly divided into two proximal (P1 and P2) and two distal (D1 and D2) sections, with the proximal sections approximately corresponding to the jejunum and the distal ones to the ileum. P2 and D2 were fixed in 4% paraformaldehyde overnight and subsequently embedded in paraffin. Hematoxylin and eosin (H&E) staining of 4 μm thick sections were evaluated microscopically using a 0–5 grading system published previously [26], which is shown in Figure 1 and described in the Results section. Using ImageScope (Aperio, Vista, USA), five random fields of view (100 × magnification) were taken and each individual villus was graded (~50 total villi per intestinal region). The assessor was blinded to the treatment groups.

### Malondialdehyde (MDA) assay

The P1 and D1 sections of the intestines were collected post-reperfusion and flushed with ice-cold PBS before being snap frozen in liquid nitrogen and stored at −80°C. For the assay, tissue was mechanically homogenized and processed using an Ultra-Turrax disperser (IKA, Guangzhou, China). An OxiSelect Thiobarbituric Acid Reactive Substances (TBARS) Assay Kit (Cell Biolabs Inc, San Diego, USA) was used to determine the MDA content in the supernatants of the homogenates via colorimetric quantification (532 nm) of the MDA-thiobarbituric acid reaction. Total protein concentration (t.p.) was determined using the Bio-Rad Protein Assay (Bio-Rad, Hercules, USA). Absolute MDA values were normalized to their respective individual total protein concentrations by dividing absolute MDA value by total protein concentration. Results are expressed as nmol MDA/mg total protein.

### Determination of cytokines

After reperfusion, blood was collected into heparinized tubes and plasma isolated via centrifugation (300 × g for 10 min). Plasma was snap frozen in liquid nitrogen and stored at −20°C until assayed. Plasma IL-6 was measured by ELISA using the mouse IL-6 ELISA Set (BD Biosciences, San Diego, USA).

### Statistical analysis

Data analysis was performed using GraphPad Prism 5.0 (GraphPad Software, San Diego, USA). The difference in frequency of histopathological grades between treatment groups was analyzed by chi-square test. All other comparisons between groups were calculated using the Mann–Whitney rank sum *U*-test (one tailed). Differences were considered to be statistically significant if the *P* value was less than 0.05. All data are presented as means ± standard error of the mean (s.e.m.).

## Results

### Animals preconditioned with remifentanil exhibit significantly reduced mucosal damage

Using the scoring system published by Chiu et al. [26], we evaluated the severity of mucosal injury to the intestines after IRI using a numerical grading score of 0 to 5 as shown in Figure 1. Grade 0 represents normal villi and grades 1–5 indicate increments of severity of injury from mild (1), identified by development of subepithelial Gruenhagen’s spaces (Figure 1, black arrow), to progressive lifting of the epithelial layer from the lamina propria (moderate - grade 2, severe - grade 3). Completely denuded villi (grade 4) and disintegration of the lamina propria (grade 5) is characteristic of the severe end of the spectrum of intestinal IRI.

**Figure 2:**
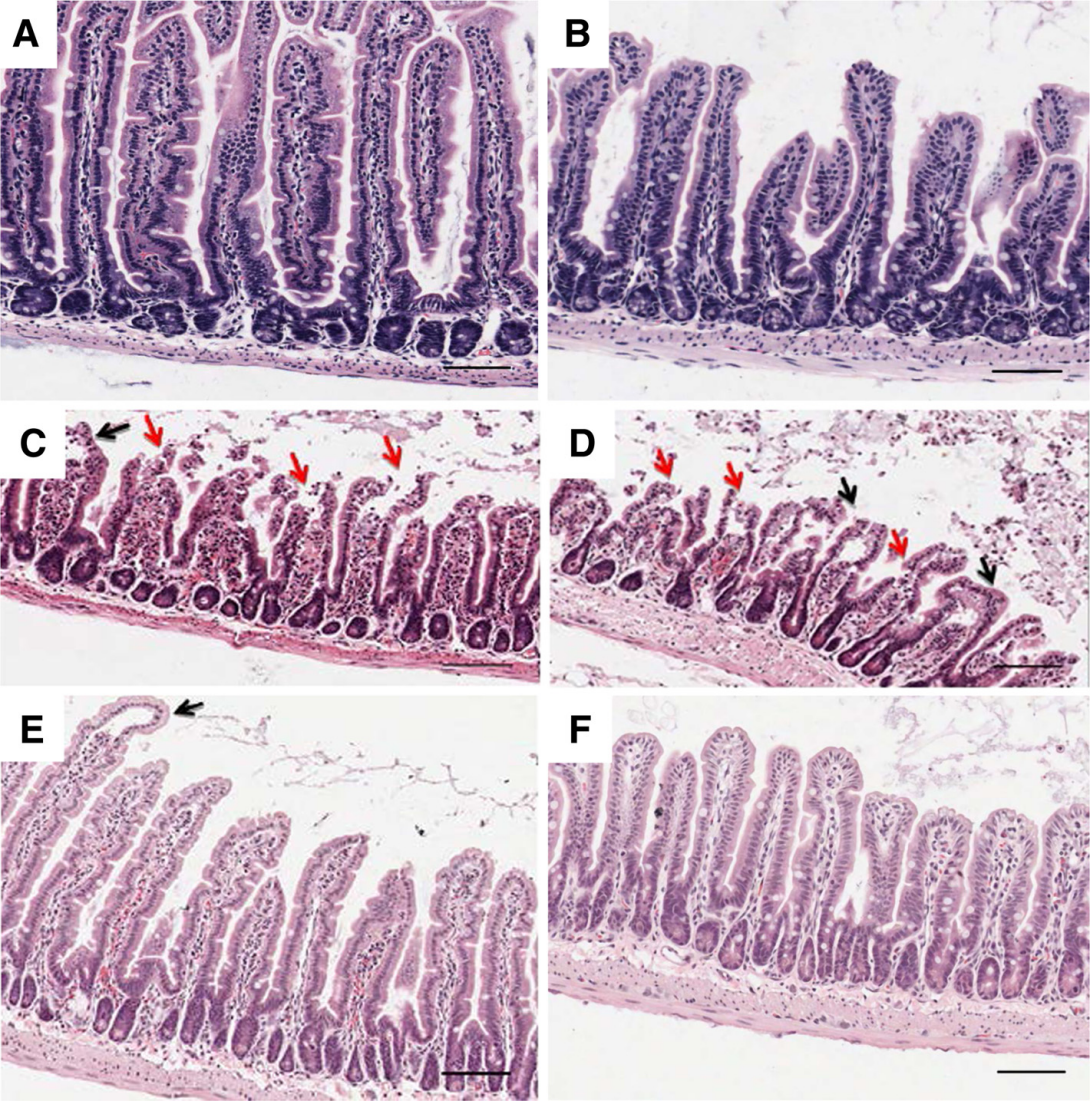
**Effect of remifentanil pretreatment on the jejunum and the ileum after ischemia-reperfusion or sham laparotomy.** Photomicrographs depicting H&E-stained intestinal sections of mice pretreated with saline (**C** jejunum, **D** ileum) or remifentanil (**E** jejunum, **F** ileum) after ligation of the superior mesenteric artery for 30 min and reperfusion of 60 min. Representative photomicrographs of remifentanil or saline pretreated sham-operated controls are also displayed (**A** jejunum, **B** ileum). n = 9 for saline and n = 7 for remifentanil (IR group); n = 4 for each treatment (Sham group). Red arrows point to severe injury with denuding of villi tips (grade 3). Black arrows indicate Gruenhagen’s spaces, a sign of mild ischemia-reperfusion injury (grade 1). Magnification 100 ×; scale bars indicate 100 μm.

Figure 2 depicts representative images of intestines from mice of the different treatment groups, with the sections of the jejunum (proximal intestine) in the left column (panels A, C, E) and the ileum (distal intestine) in the right column (panels B, D, F). Mice in the remifentanil preconditioning (RPC) group (Figure 2E, F) displayed improved preservation of healthy gut mucosa after reperfusion compared to saline-pretreatment (Figure 2C, D). No histological differences were observed between the treatment groups (RPC or saline) in the sham-operated animals (Figure 2A, B). As expected, the overall health of the intestinal mucosa was nearly unaffected in this sham control group.

**Figure 3:**
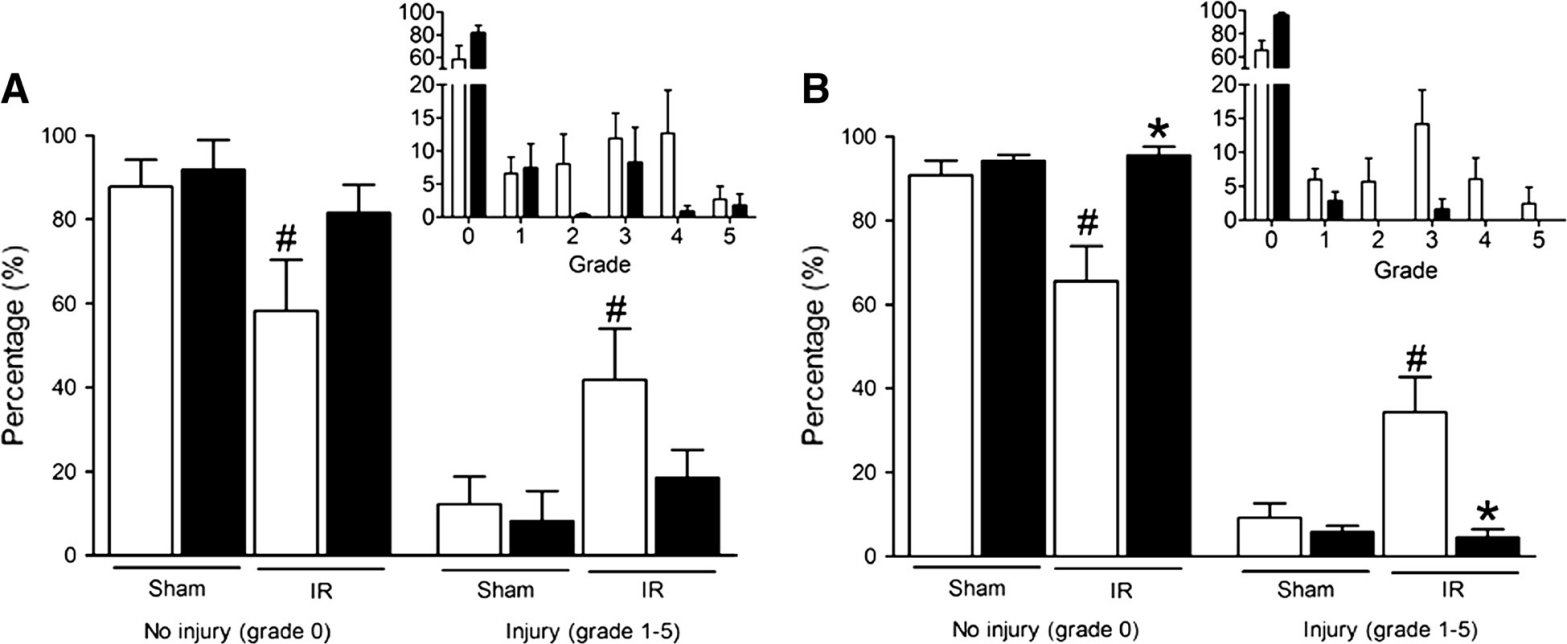
**Grading of the injury in the small intestine after ischemia-reperfusion or sham operation.** No injury was defined as grade 0; increasing severity of injury was graded from 1 to 5 as described in Figure 1. The presence or absence of mucosal injury in the jejunum (**A**) and ileum (**B**) after sham operation (Sham) or ischemia-reperfusion (IR) is shown. Open bars indicate saline pretreatment, remifentanil-preconditioning is represented by the filled bars. The insets display the percentage for each of the grades in the IR set. Chi-square analysis demonstrated significantly more damaged villi in the saline pretreated mice than the RPC animals (*P* < 0.001). Results are shown as means of percentages ± s.e.m; *, *P* < 0.05 for saline vs remifentanil; #, *P* < 0.05 for sham (treatment groups combined) vs IR. n = 9 for saline and n = 7 for remifentanil (IR group); n = 4 for each treatment (Sham group).

As illustrated in Figure 3A, significant injury as a result of IRI in the jejunum was evident by the increase in percentage of injured villi in saline-pretreated mice in comparison to the two sham-operated groups (percentage for grades 1-5: 42% for IR-saline vs 12% for sham-saline and 8% for sham-RPC). On the other hand, the percentage of injured villi in the RPC group was comparable to that in the sham-operated groups (18% grade 1-5). Among the sham groups, no differences were observed in any of the parameters we investigated.

Using chi-square analysis on the distribution of histological injury grades (Figure 3 insets), we observed that there was a significant difference in the degree of injury between RPC and saline-pretreated mice post-IRI (*P* < 0.001). In the saline-pretreated group post-IRI, grade 3 (12%) and 4 (13%) mucosal injury was the most common finding, whereas in the RPC animals, the pattern of injury we observed was milder, with the majority of villi falling into grades 1–3 (16%).

Although this trend towards amelioration of IRI by remifentanil in the jejunum did not reach statistical significance (*P*=0.12), it was consistent with the impressive findings we obtained from samples of the ileum (Figure 3B). The ileum of RPC animals post-IRI exhibited an almost completely healthy mucosa, unlike that of saline-pretreated animals (96% vs 66% grade 0). Among the remaining 4% of injured mucosa in the RPC group, nearly all villi were scored grade 1, whereas villi from saline-pretreated animals were scored across the entire spectrum of severity (grades 1–5, Figure 3B inset). The ileal mucosa in the RPC group after IRI was identical to that in sham-operated animals (91% and 94% grade 0 for RPC and saline), both on the overall level (Figure 3B) and the individual grades of injury (exclusively grade 1 in the sham groups, which are not shown in the insets).

### Preconditioning with remifentanil reduces oxidative stress in intestinal ischemia-reperfusion injury

**Figure 4:**
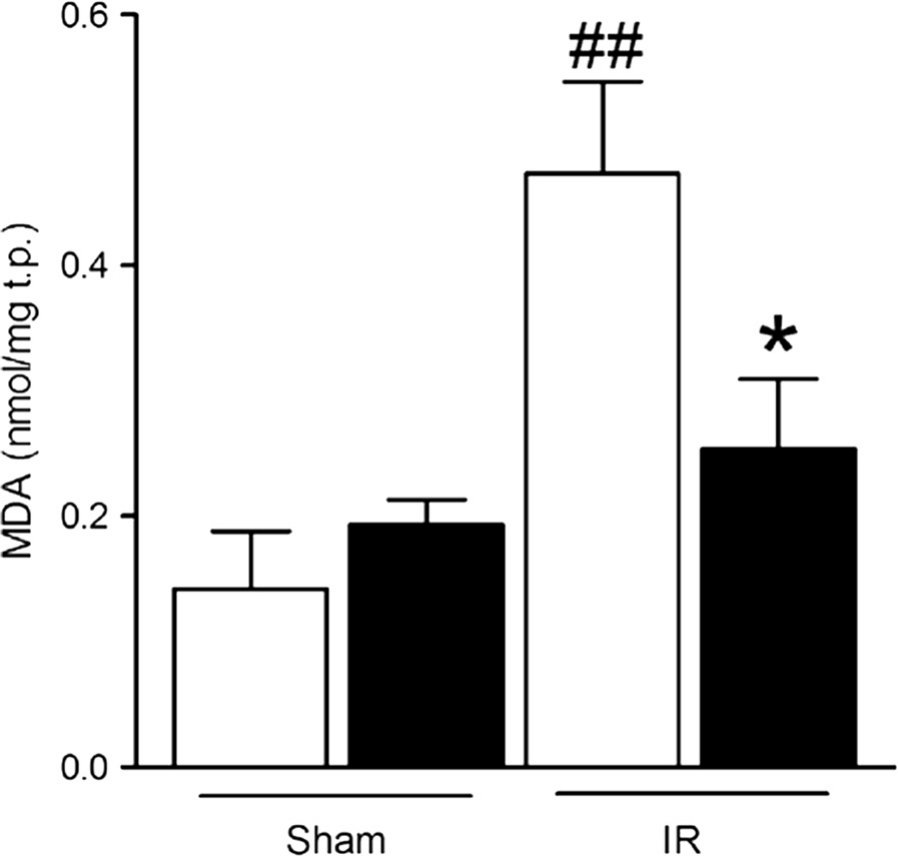
**Effect of remifentanil preconditioning on lipid peroxidation in intestinal tissue after ischemia-reperfusion-induced injury or sham laparotomy.** Tissue homogenates of mouse ileum post ischemia-reperfusion (IR) and sham-operated animals (Sham) were assayed for MDA content. All MDA values are normalized to the individual total protein (t.p.) content in the respective sample. Open bars, saline pretreatment; filled bars, remifentanil pretreatment. Bars show normalized concentrations (means ± s.e.m); *, *P* < 0.05 for saline vs remifentanil; ##, *P* < 0.01 for Sham (treatment groups combined) vs IR. n = 4 for saline and 6 for remifentanil (IR groups); n = 3 for saline and 4 for remifentanil (Sham groups).

Homogenates of the jejunum and the ileum were assayed for MDA as a marker of oxidative stress [27]. The abundance of MDA in the ileum (Figure 4) was significantly elevated in saline-pretreated mice after IR in comparison with the sham-operated groups (0.47 nmol/mg t.p. in IR-saline vs 0.14 and 0.19 nmol/mg t.p. in sham-saline and -RPC). Consistent with the histological findings, RPC protected the ileal tissue from this increase in lipid peroxidation (MDA at 0.25 nmol/mg t.p. after IR and remifentanil, i.e. nearly at steady state levels).

However, this pattern was different in the jejunum, where we measured considerably more MDA under sham conditions (0.47 ± 0.05 and 0.40 ± 0.09 nmol/mg t.p. for saline and RPC). As in each of the other parameters of IRI, there was no difference between RPC and saline pretreatment; however, the difference between the jejunum and the ileum was significant (*P* < 0.01 for both sham groups in the jejunum vs both sham groups in the ileum). In mice that had undergone intestinal IRI and saline pretreatment, the jejunal abundance of MDA was similar (0.41 ± 0.04 nmol/mg t.p.) and RPC also did not significantly change lipid peroxidation in this section of the intestine (0.36 ± 0.07 nmol/mg t.p.).

### The induction of IL-6 is attenuated by pretreatment with remifentanil

**Figure 5:**
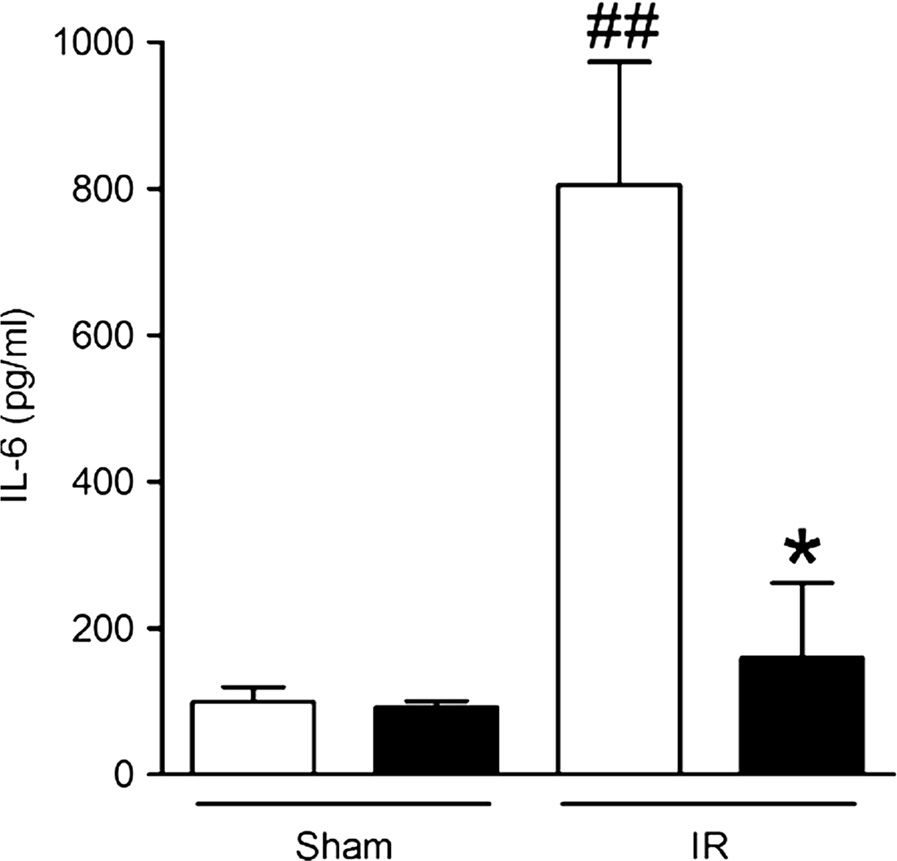
**Plasma IL-6 abundance post ischemia-reperfusion or sham laparotomy in remifentanil-pretreated and saline control mice.** Blood was obtained immediately after the 60 min period of reperfusion and IL-6 was assayed by ELISA. Open bars, saline pretreatment; filled bars, remifentanil pretreatment. Bars show absolute cytokine concentrations (means ± s.e.m.) in the sham (Sham) and ischemia-reperfusion (IR) groups; *, *P* < 0.05 for saline vs remifentanil; ##, *P* < 0.01 for Sham (combined treatment groups) vs IR. n = 4 for both saline groups and the remifentanil/Sham group, n = 5 for the remifentanil/IR group.

As expected, the abundance of IL-6 in the plasma of sham-operated mice was low and there was no difference between the pretreatment groups (Figure 5). IRI induced a marked, approximately 8-fold increase of plasma IL-6 (to 805 pg/ml), which was reduced to 159 pg/ml, i.e. nearly to the level of the sham groups, by preconditioning with remifentanil.

## Discussion

We have shown that preconditioning with remifentanil powerfully counteracts the injury to the intestinal mucosa caused by a period of ischemia and subsequent reperfusion. A marked amelioration of mucosal injury in remifentanil-treated mice was accompanied by a reduction of oxidative stress locally and inflammation systemically, as evidenced by decreased concentrations of gut tissue MDA and plasma IL-6. Although morphine has been shown to precondition the rat intestine, our study is first to demonstrate that remifentanil conveys a similar protective effect, possibly through an attenuation of systemic inflammation in addition to triggering the preconditioning cascade [28]. This has important potential clinical implications because, unlike morphine, remifentanil can be given in high doses intraoperatively without the risk of residual postoperative adverse effects such as respiratory depression and gut dysmotility [29].

Remifentanil is administered to patients as a bolus or as a continuous intravenous infusion. Both a preconditioning bolus as well as a continuous infusion has been found to reduce myocardial infarction to a similar extent [20]. However, the preconditioning bolus was observed to be better at preserving myocardial function, suggesting that bolus administration may be superior to continuous infusion. A single bolus injection can be easily managed and allows for higher doses and thus higher peak levels, which likely boost protective effects. Indeed, in myocardial models of IRI, an increased RPC dosage has been shown to reduce infarct size and the abundance of pro-inflammatory mediators such as IL-6 in a dose-dependent manner [30].

Both the production of reactive oxygen species (ROS), as well as increased apoptosis contribute significantly to epithelial damage in intestinal IRI [3,31]. Mechanically-induced ischemic preconditioning of the heart [32] and intestine [33] has been shown to increase expression of Bcl-2, which has an anti-apoptotic as well as anti-oxidant property. Similarly, in remifentanil-induced heart preconditioning, the expression of Bcl-2 is also reported to increase. Moreover, the expression of Bax, a promoter of ROS production as well as apoptosis, is reduced [23,34]. Therefore, the prevention of the IRI-induced increase in lipid peroxidation and thus MDA by remifentanil may in part be due to increased Bcl-2 or reduced Bax activity, which in turn may contribute to the reduction of tissue injury and apoptosis.

Interestingly, remifentanil pretreatment significantly decreased ischemia-reperfusion-induced oxidative stress in the ileum, but not in the jejunum. This difference in the pattern of MDA distribution between the proximal and the distal sections of the gut suggests that jejunum and ileum are not identical with regard to lipid peroxidation and in their response to oxidative stress. Indeed, such differences have been observed by others. For instance, in a study of LPS-induced intestinal injury, lipid peroxidation increased only in the ileum, not in the jejunum [35]. Another study that investigated pharmacological preconditioning with isoflurane in intestinal IRI also reported protection only of the ileum, not the jejunum, thus concurring with our findings [36]. On the other hand, in our opinion, it is rather unlikely that there was no difference in oxidative stress at any time, as IRI was histologically similar between the jejunum and ileum (i.e. comparing the sham groups with the IR-saline groups in both sections of the gut). It is possible that the timepoint at which we sampled the gut was not optimal to show differences that may occur at an earlier or later time.

Inflammation is another important contributor to the aggravation of IRI [19,37], and we measured IL-6 as a marker of the inflammatory response [18,38]. The ability of remifentanil to reduce IL-6 has been previously reported in coronary artery bypass graft surgery and open cholecystectomy [39,40]; however, reports on this effect in the context of IRI are not abundant. One study demonstrated that RPC reduced systemic IL-6 in IRI of the heart [41], which is consistent with the data we report here. The observation that RPC reduces systemic IL-6 after intestinal IRI is remarkable, as the single-dose RPC strategy protected not only the organ affected by the ischemic insult, but also blunted the systemic inflammatory response. RPC may thus confer a potential benefit regarding the systemic consequences of intestinal injury, which are often severe and can include sepsis, multi-organ failure and even death [42-45].

Opioids have been used as analgesics for centuries but it is only in the last decade or so that they have been discovered to also play a significant role in immunomodulation. Although the immunomodulatory effects of remifentanil are not as well studied as those of morphine and fentanyl, current data suggest that its receptor-mediated effects are not different from other μ-opioid agonists [46]. Immune cells express functionally active opioid receptors on their surface, and opioids have been shown to exert their effect directly via these receptors [47,48]. Such effects include the inhibition of the activity of nuclear factor-kappaB (NF-κB), the prototypical upstream facilitator of IL-6 production [49]. For example, chronic morphine treatment significantly impaired the translocation of the NF-κB p65 and p50 transcription factors into the nuclear compartment of a variety of cells in a model of lung infection [50]. Moreover, the opioid-induced preconditioning signaling cascade has been reported to activate the phosphatidylinositol-3-kinase/Akt and the three mitogen-activated protein kinase (MAPK) pathways c-Jun N-terminal kinase (JNK), p38 MAPK, and extracellular signal-regulated kinase (ERK) 1/2. Activation of these signaling cascades in preconditioning has been linked to pro-survival and anti-apoptotic effects [51-57]. Furthermore, it is possible that such mild stimulation of inflammatory pathways induces counter-regulatory mechanisms such as the release of anti-inflammatory cytokines and preconditions cells to reduce the impact of a stronger consecutive stimulus. Such conditioning cascades have been described for low doses of LPS that protect against subsequent IRI [58,59].

## Conclusion

Intestinal IRI is an important source of morbidity in a number of clinical situations such as cardiac surgery, vascular surgery, organ transplantation as well as neonatal diseases such as necrotizing enterocolitis and persistent ductus arteriosus. Our study has demonstrated that a single bolus of remifentanil given before tissue ischemia protects against IRI in the small intestine, allowing us to bypass the inherent adverse effects of conventional μ-opiods such as persistent inhibition of gastrointestinal motility and respiratory drive. Based on these findings, remifentanil is worthy of further mechanistic and clinical investigation as a potential novel pharmacological intervention strategy in ischemia-reperfusion of the intestine and other organs.

## Abbreviations

H&E: Hematoxylin and eosin; IL-6: Interleukin- 6; IRI: Ischemia-reperfusion injury; MAPK: Mitogen-activated protein kinase; MDA: Malondialdehyde; NF-κB: Nuclear factor kappaB; PBS: Phosphate buffered saline; ROS: Reactive oxygen species; RPC: Remifentanil preconditioning; s.e.m.: Standard error of the mean; SMA: Superior mesenteric artery; t.p.: Total protein.

## Competing interests

The authors declare that they have no competing interests.

## Authors’ contributions

SSCC, MGI, WC and CANP designed the study; SSCC, IR, WC and CANP performed the experiments; SSCC, IR, MFN, WC and CANP analyzed the data; SSCC, PJB, MFN, MGI, WC and CANP wrote the manuscript. All authors read and approved the final manuscript.

## Pre-publication history

The pre-publication history for this paper can be accessed here:

http://www.biomedcentral.com/1471-230X/13/69/prepub
